# Drug-induced dermatomyositis: a pharmacovigilance study of the FDA adverse event reporting system

**DOI:** 10.3389/fphar.2025.1526836

**Published:** 2025-10-01

**Authors:** Jianing Bi, Hanzhang Xie, Yixuan Yang, Qiaochu Chen, Bingnan Cui, Zhanshuo Xiao

**Affiliations:** ^1^ Department of Dermatology, Guang’anmen Hospital, China Academy of Chinese Medical Sciences, Beijing, China; ^2^ College of Traditional Chinese Medicine, Beijing University of Chinese Medicine, Beijing, China

**Keywords:** adverse events, dermatomyositis, drug-induced dermatomyositis, FAERS, pharmacovigilance

## Abstract

**Background:**

Dermatomyositis (DM) is an autoimmune disease that may be triggered by certain medications. However, most studies have focused on specific drugs, lacking a comprehensive overview. This study uses the FDA’s Adverse Event Reporting System (FAERS) to explore the correlation between DM and medications.

**Research design and methods:**

The study encompassed FAERS reports from January 2004 to June 2024. We coded and classified adverse events (AEs) using MedDRA and conducted multiple disproportionality analyses (ROR, PRR, BCPNN, MGPS) to examine drug-event associations and analyze the results.

**Results:**

Using the “primary suspects” role code in FAERS, 1767 reports involving 353 drugs suspected of inducing DM were identified. Among 24 signal-positive drugs, cardiovascular drugs (297 reports, mainly statins) were most frequent, followed by immunotherapy agents (188 reports, mainly Immune Checkpoint Inhibitors) and chemotherapy agents (147 reports, mainly Antimetabolites).

**Conclusion:**

This study on drug-induced DM presents a new approach to rational and evidence-based drug prescribing. It leverages advanced model algorithms to significantly improve the precision in predicting drug-DM correlations, enhancing patient safety. Additionally, the study provides clinicians with guidance on avoiding medications associated with DM in patients with predisposing factors that may increase their risk of developing the condition.

## 1 Introduction

Dermatomyositis (DM) is an idiopathic inflammatory myopathy that mainly affects the skeletal muscles and skin, clinically manifested as symmetrical proximal muscle weakness and myalgia, as well as typical skin lesions such as periorbital purpuric edema patches, neck and chest congestive macules, Gottron papules, and so on DeWane et al. (2020) and McKee et al. (2024). Epidemiological research estimates that the incidence of DM can be as high as 1.1 per 100,000 person-years, showing an upward trend (Kronzer et al., 2023). Additionally, the male-to-female ratio is 1:2. Clinically, the diagnosis of DM is established through a comprehensive assessment encompassing cutaneous manifestations, systemic symptoms, abnormalities in serum muscle enzymes or autoantibody levels, muscle testing for power, electromyography findings, magnetic resonance imaging and pathological biopsy results. However, in the absence of characteristic cutaneous or muscular manifestations, the diagnosis of DM may be elusive, and current data indicate that over 20% of adult DM patients exhibit clinically amyopathic DM, suggesting that the prevalence of DM may be higher than reported in existing studies (Bendewald et al., 2010).

The etiology of DM remains elusive, with several postulated contributing factors including genetics, infection, autoimmune responses, and malignancies (Sunderkötter et al., 2016). Certain medications, including penicillamine (Solanki et al., 2024), hydroxyurea (Caravan et al., 2024), lipid-lowering drugs (Borges et al., 2018), have been implicated in triggering DM by inducing abnormal immune reactions. This entity, known as drug-induced dermatomyositis (DIDM), pertains to instances of DM that exhibit a temporal association with exposure to specific medications and cannot be attributed to any other specific underlying cause. In simpler terms, DM occurs after a patient is exposed to certain drugs and may either spontaneously resolve or necessitate ongoing treatment upon drug withdrawal. Importantly, there is a risk of recurrence upon re-exposure to the same medications. Therefore, monitoring the medication treatment process can potentially reduce the incidence of DM to a certain extent.

The Food and Drug Association’s Adverse Event Reporting System (FAERS) is the world’s largest repository of reported hazardous drug events aimed at monitoring drug safety, which provides public access to drug information, including names, active ingredients, routes of administration, and their reported roles in events. It aggregates reports submitted globally by drug regulatory authorities, medical institutions, researchers, or individual patients, with a significant contribution from the United States, encompassing adverse reactions, usage patterns, and patient data. After being reviewed and processed, these reports are assigned codes and categorized accordingly (Yu et al., 2021). Additionally, it recognizes distinct role codes for drugs, categorizing them as primary suspects, secondary suspects, interacting agents, and concomitant medications. To our knowledge, research gaps exist in the study of drugs that induce DM using FAERS. Accordingly, the primary aim of this study is to assess the reported associations between medications and DM by conducting a signal mining analysis utilizing data from the FAERS database.

## 2 Materials and methods

### 2.1 Data source

FAERS data from January 2004 to June 2024 were included in this retrospective pharmacovigilance study. The FAERS database encompasses seven distinct datasets: patient demographics (DEMO), drug information (DRUG), indications (INDI), adverse events (REAC), reporter source (RPSR), patient outcomes (OUTC), and therapy start/end dates (THER). Due to updates and resubmissions, duplicate reports exist. This study adhered to FDA guidelines, eliminating duplicates based on case number, date, and serial order, ensuring inclusion of only the most recent submissions.

### 2.2 Study design

In this study, we scrutinized the REAC files using the comprehensive Medical Dictionary for Regulatory Activities Terminology (MedDRA version 26.1), specifically focusing on the preferred term “Dermatomyositis” from the Important Medical Events (IME) terminology list, to accurately identify cases of DM, including those cases involving juvenile dermatomyositis (JDM). Given the concurrent use of multiple drugs among most patients, the potential causative drugs associated with reported adverse events (AEs) in the DRUG dataset are categorized as primary suspect (PS), secondary suspect, interacting, and concomitant drugs. This study exclusively included drugs labeled as “PS” for DM AEs. As different roles can be chosen by the submitter for the reported drug, we standardized the initial drug names (brand, generic, synonyms, abbreviations) to generic forms using search strategies and resources like DrugBank, FDA, and PubMed. Various disproportionality analyses, including Reporting Odds Ratio (ROR), Proportional Reporting Ratio (PRR), Bayesian Confidence Propagation Neural Network (BCPNN), and multi-item gamma Poisson shrinker (MGPS), were used to identify the association between drugs and AEs.

### 2.3 Statistical analysis

A descriptive analysis of DM cases encompassed demographics, reporting regions, reporters, annual trends, and outcomes. A case-control analysis of the top 50 drugs ranked by the number of reports was performed using a 2 × 2 contingency table, with four algorithms utilized to calculate drug-specific values. The specific contents of the four algorithms are shown in Supplementary Material. Drugs that concurrently meet the criteria of four algorithms are deemed significant positive signals. Those that solely fulfill the ROR algorithm are considered potentially correlated and still warrant further clinical attention and vigilance. All data processing and statistical analyses were performed using R software (version 4.4.1).

## 3 Result

### 3.1 Characteristics of the FAERS study population

Between January 2004 and June 2024, our study extracted a total of 21,433,114 AE reports from the FAERS database. After removing 3,250,202 duplicate reports, 18,182,912 unique adverse event reports were obtained. Ultimately, 1767 reports related to DM were identified. The clinical characteristics and report information are summarized in Table 1. In the analyzed reports, there was a slight female predominance (56.6%) compared to males (34.2%). In terms of weight, patients weighing 50–100 kg accounted for 18.0%. Regarding age, the majority of patients were in the 41–65 age range (37.4%), followed by those aged 66–85 years (24.1%). The majority of reports were submitted by Physicians (44.9%) and consumers (16.2%). Geographically, the United States accounted for the highest proportion of reports (26.4%), followed by Japan (13.0%), Canada (8.4%), France (7.1%) and United Kiongdom (5.4%). Regarding outcomes, the majority of reports indicated other serious outcomes (42.7%), followed by hospitalization (37.1%) and death (9.0%). The most frequently reported indications were Rheumatoid arthritis (6.8%), Dyslipidaemia (3.3%), Hypercholesterolaemia (3.0%), Breast cancer (2.5%), and Psoriasis (1.9%). The highest number of cases recorded within the report profile occurred in 2021 (n = 206, 11.7%), followed by 2023 (n = 188, 10.6%). Lastly, regarding the time of occurrence from drug administration to disease onset, most reports (75%) lack precise documentation. Specifically, 9% of the reports indicate a duration of >1 month to ≤1 year, followed by 6% reporting less than 1 week. Both durations of >1 week to <1 month and >1 year are documented in 5% of the reports. In summary, the time of occurrence from drug administration to disease onset is a complex interplay of multiple factors, including the pharmacokinetic properties of the drug, individual variability in drug response, dosage and frequency of administration, interaction with other drugs or substances, disease state and severity, and route of administration. Therefore, it is crucial to monitor patients closely for any adverse reactions after drug administration and to adjust the treatment plan accordingly.

**TABLE 1:** Demographic and clinical characteristics of DIDM cases from the FAERS.

Characteristics	N (%)	Reporting year	N (%)
**Sex**		2004	40 (2.3%)
F	1,001 (56.6%)	2005	50 (2.8%)
M	604 (34.2%)	2006	42 (2.4%)
Missing	162 (9.2%)	2007	49 (2.8%)
**Weight (kg)**		2008	43 (2.4%)
<50	46 (2.6%)	2009	54 (3.1%)
>100	32 (1.8%)	2010	77 (4.4%)
50∼100	318 (18.0%)	2011	26 (1.5%)
Missing	1,371 (77.6%)	2012	42 (2.4%)
**Age (year)**		2013	63 (3.6%)
<18	45 (2.5%)	2014	67 (3.8%)
>85	11 (0.6%)	2015	82 (4.6%)
18–40	194 (10.9%)	2016	54 (3.1%)
41–65	662 (37.4%)	2017	76 (4.3%)
66–85	426 (24.1%)	2018	102 (5.8%)
Missing	429 (24.3%)	2019	103 (5.8%)
**Reported Person**		2020	130 (7.4%)
Consumer	287 (16.2%)	2021	206 (11.7%)
Health Professional	265 (15.0%)	2022	148 (8.4%)
Lawyer	8 (0.5%)	2023	188 (10.6%)
Physician	793 (44.9%)	2024	125 (7.1%)
Other health-professional	261 (14.8%)		
Pharmacist	65 (3.7%)		
Missing	88 (5.0%)		
Reported Countries (top five)			
United States	466 (26.4%)		
Japan	230 (13.0%)		
Canada	149 (8.4%)		
France	125 (7.1%)		
United Kiongdom	96 (5.4%)		
Outcome			
Death	159 (9.0%)		
Life-Threatening	52 (2.9%)		
Hospitalization	656 (37.1%)		
Disability	38 (2.2%)		
Other Serious	755 (42.7%)		
Missing	107 (6.1%)		
Indications (top five)			
Rheumatoid arthritis	120 (6.8%)		
Dyslipidaemia	59 (3.3%)		
Hypercholesterolaemia	53 (3.0%)		
Breast cancer	45 (2.5%)		
Psoriasis	33 (1.9%)		
Time of occurrence			
≤ l week	101 (6%)		
>l week,≤ l month	88 (5%)		
>l month,≤ l year	164 (9%)		
>l year	86 (5%)		
Unknown	1,328 (75%)		

### 3.2 Characteristics of signaling drugs

Out of the 1767 adverse event (AE) reports linked to DM identified in this study, a total of 578 unique drug names were listed as “PS.” By consolidating these names, which encompassed both brand and generic versions, we identified 354 distinct medications. The top 50 DM-related drugs with the highest number of reports are shown in Table 2, with those fulfilling the criteria of the ROR method indicated in bold. Atorvastatin topped the list with 171 reports, followed by Adalimumab (n = 81), Nivolumab (n = 72), Etanercept (n = 59), Methotrexate (n = 52) and Rosuvastatin (n = 50). To provide a more precise elucidation of the relationship between medications and AEs associated with DM, we conducted a further analysis of drugs that fulfilled all four disproportionality analysis methods. Among the top 50 reports ranked by quantity, a comprehensive analysis revealed a total of 24 distinct drugs that were significantly associated with DM. These drugs concurrently met all four disproportionality analysis methods, and the detailed findings are presented in Table 3. Among the 24 drugs exhibiting significant associations, we observed a diverse array of drug classes, specifically cardiovascular drugs (n = 297), immunotherapy agents (n = 188), chemotherapy agents (n = 147), antibiotics (n = 33), immunosuppressants (n = 17), and proton pump inhibitors (n = 17). It is noteworthy that Hydroxyurea, Fenofibrate, and Atorvastatin demonstrated significantly elevated RORs of 41.66, 24.49, and 24.14, respectively, suggesting a robust connection to AEs associated with DM. Atezolizumab had an ROR of 17.02, emphasizing the need for safety monitoring. Although Infliximab (n = 28, ROR 1.03) and Human Immunoglobulin G (n = 15, ROR 1.47) did not fulfill all four disproportionality criteria, the relatively high number of reports associated with these drugs suggests that further clinical monitoring may be warranted. Despite the stability and reliability of four disproportionality analyses, drugs with a high number of real-world AE reports meeting even one statistical method still require further clinical attention and vigilance.

**TABLE 2:** The top 50 DM-related drugs with the highest number of reports.

Drug	n	ROR
Atorvastatin	171	**24.14**
Adalimumab	81	**1.3**
Nivolumab	72	**12.87**
Etanercept	59	**1.36**
Methotrexate	52	**3.12**
Rosuvastatin	50	**11.91**
Simvastatin	43	**13.35**
Trastuzumab	36	**8.77**
Docetaxel	34	**5.87**
Pembrolizumab	33	**7.19**
Tofacitinib	31	**2.32**
Atezolizumab	30	**17.02**
Capecitabine	29	**5.63**
Infliximab	28	1.03
Rituximab	24	**1.52**
Carboplatin	23	**5.27**
Paclitaxel	22	**6.4**
Ipilimumab	17	**11.05**
Omeprazole	17	**4.23**
Doxycycline	17	**11.84**
Hydroxychloroquine	17	**8.18**
Amlodipine	15	**2.33**
Human Immunoglobulin G	15	1.47
Hydroxyurea	**14**	**41.66**
Denosumab	12	1.16
Alendronic Acid	14	1.37
Bevacizumab	**13**	**1.92**
Fenofibrate	**13**	**24.49**
Ramipril	**13**	**5.86**
Zoledronic Acid	13	1.6
Cyclosporine	**13**	**2.69**
Interferon beta-1a	11	0.71
Clarithromycin	**9**	**5.01**
Gemcitabine	**9**	**3.53**
Golimumab	**9**	**2.47**
Anastrozole	**8**	**4.58**
Apremilast	8	0.97
Olaparib	**8**	**7.79**
Palbociclib	8	0.89
Tacrolimus	8	1.33
Celecoxib	**8**	**2.3**
Imatinib	8	1.55
Dalfampridine	7	1.25
Certolizumab pegol	7	0.93
Ezetimibe	**7**	**10.26**
Letrozole	**7**	**2.6**
Minocycline	**7**	**13.4**
Tocilizumab	7	0.81
Niraparib	7	1.44
Abatacept	6	0.72

*Those fulfilling the criteria of the ROR method are indicated in bold.

**TABLE 3:** Signaling drugs of drug-associated DM cases from the FAERS.

Drug	n	ROR (95%Cl)	PRR (χ^2^)	EBGM (EBGM05)	IC (IC025)
Chemotherapy agents (n = 147)					
Antimetabolites (n = 60)					
Capecitabine	29	5.63 (3.9–8.13)	5.63 (108.64)	5.55 (4.09)	2.47 (0.81)
Gemcitabine	9	3.53 (1.83–6.79)	3.53 (16.22)	3.52 (2.03)	1.81 (0.15)
Olaparib	8	7.79 (3.89–15.6)	7.79 (47.12)	7.76 (4.34)	2.96 (1.29)
Hydroxyurea	14	41.66 (24.61–70.51)	41.6 (550.42)	41.28 (26.58)	5.37 (3.7)
Taxanes (n = 56)					
Paclitaxel	22	6.4 (4.2–9.74)	6.4 (98.96)	6.33 (4.45)	2.66 (1)
Docetaxel	34	5.87 (4.18–8.24)	5.87 (134.65)	5.77 (4.35)	2.53 (0.86)
Platinum compounds (n = 23)					
Carboplatin	23	5.27 (3.49–7.95)	5.27 (78.45)	5.21 (3.69)	2.38 (0.71)
Aromatase inhibitors (n = 8)					
Anastrozole	8	4.58 (2.29–9.18)	4.58 (22.32)	4.57 (2.55)	2.19 (0.52)
Immunotherapy agents (n = 188)					
Immune checkpoint inhibitors (n = 152)					
Atezolizumab	30	17.02 (11.86–24.42)	17.01 (444.46)	16.74 (12.38)	4.07 (2.4)
Nivolumab	72	12.87 (10.17–16.3)	12.87 (756.56)	12.39 (10.17)	3.63 (1.96)
Pembrolizumab	33	7.19 (5.09–10.14)	7.18 (172.42)	7.07 (5.3)	2.82 (1.15)
Ipilimumab	17	11.05 (6.85–17.82)	11.05 (153.85)	10.95 (7.34)	3.45 (1.79)
Monoclonal antibodies (n = 36)					
Trastuzumab	36	8.77 (6.31–12.21)	8.77 (242.93)	8.62 (6.54)	3.11 (1.44)
Antibiotics (n = 33)					
Tetracyclines (n = 24)					
Minocycline	7	13.4 (6.38–28.15)	13.39 (79.95)	13.34 (7.17)	3.74 (2.07)
Doxycycline	17	11.84 (7.34–19.09)	11.84 (167.04)	11.73 (7.87)	3.55 (1.89)
Macrolides (n = 9)					
Clarithromycin	9	5.01 (2.6–9.64)	5.01 (28.7)	4.99 (2.88)	2.32 (0.65)
Immunosuppressants (n = 17)					
Disease-modifying antirheumatic drugs (n = 17)					
Hydroxychloroquine	17	8.18 (5.07–13.19)	8.18 (106.06)	8.11 (5.44)	3.02 (1.35)
Cardiovascular drugs(n = 297)					
Statins (n = 264)					
Atorvastatin	171	24.14 (20.62–28.26)	24.12 (3,428.63)	21.92 (19.21)	4.45 (2.79)
Simvastatin	43	13.35 (9.87–18.07)	13.35 (479.37)	13.05 (10.13)	3.71 (2.04)
Rosuvastatin	50	11.91 (8.99–15.78)	11.91 (485.54)	11.6 (9.17)	3.54 (1.87)
Fibrates (n = 13)					
Fenofibrate	13	24.49 (14.19–42.26)	24.47 (290.5)	24.3 (15.39)	4.6 (2.94)
Cholesterol absorption inhibitors (n = 7)					
Ezetimibe	7	10.26 (4.88–21.55)	10.25 (58.23)	10.22 (5.49)	3.35 (1.69)
ACE inhibitors (n = 13)					
Ramipril	13	5.86 (3.4–10.11)	5.86 (52.01)	5.82 (3.69)	2.54 (0.87)
Proton pump inhibitors (n = 17)					
Omeprazole	17	4.23 (2.62–6.81)	4.23 (41.47)	4.2 (2.81)	2.07 (0.4)

## 4 Discussion

### 4.1 The analysis of medication

Conventionally, DM is regarded as an ischemic myopathy that arises from endothelial cell damage mediated by the complement cascade (Luo and Mastaglia, 2015). DM is caused by a combination of multiple factors, including genetic susceptibility, environmental triggers, immunological factors (Guo et al., 2024). Drugs are a pivotal causative factor in the induction of DM, potentially eliciting abnormal T-cell differentiation. This disruption may manifest as an imbalance in the ratio of Th1 and Th2 cells or dysregulation in the differentiation of Th17/Treg cells (Sun et al., 2016). Consequently, the production of cytokines such as IL-4, IL-5, IL-13, and IL-31 is augmented, intensifying humoral immune responses and B-cell activity (Zhao and Si, 2023). This heightened immune state can give rise to autoantibodies that potentially harm muscle and skin cells, contributing to the formation of myositis-specific antibodies, including anti-Mi2, anti-melanoma differentiation-associated protein 5, anti-NXP2, anti-TIF1, and anti-small ubiquitin-like modifier activating enzyme. These antibodies, in turn, can lead to capillary destruction, precipitating ischemia, microinfarction, hypoperfusion, and perifascicular atrophy, ultimately triggering the onset and progression of DM (Seidler and Gottlieb, 2008).

In this study, we amassed a dataset comprising 1767 cases from the FAERS utilizing four distinct algorithms: “ROR,” “PRR,” “BCPNN,” and “MGPS.” Through the consolidation of various trade names and generic designations that pertained to identical drugs, we derived a total of 353 unique drug entities. Notably, 24 of these drugs emerged as having significant signals, a consensus identified by all four applied algorithms.

Our study revealed that statins were the most commonly reported medications, particularly atorvastatin, rosuvastatin, and simvastatin. Onset of statins-induced DM varies, typically 2–5 years but can be as early as 3 days after starting therapy (Alvarado et al., 2015; Chemello et al., 2017). This phenomenon underscores DM risk persists regardless of statin therapy duration. Regarding the underlying mechanisms, we hypothesize that statins may promote the exposure of hidden antigens in damaged muscle cells (Fania et al., 2017). These released antigens, or possibly the drugs themselves, may be recognized as foreign substances by the immune system, triggering an immune response that primarily activates CD4^+^ T cells and results in a Th2-biased immune response (Visconti et al., 2020; Noël, 2007). This activation could potentially trigger the release of cytokines like IFN-γ and IL-17, ultimately precipitating the onset of DM. Furthermore, we propose that B cell activation may occur, leading to the production of autoantibodies targeting muscle and skin cells (e.g., anti-Mi2, anti-MDA5, anti-TIF1), which could mediate inflammation and tissue damage (Fehr et al., 2004). However, upon reviewing the literature, we identified cases where patients, despite being diagnosed with dm following biopsy and electromyography, and exhibiting elevated creatine kinase (CK) levels and positive ANA titers, tested negative for DM-specific autoantibodies. This phenomenon warrants further experimental investigation to elucidate the mechanisms underlying statin-induced DM (Visconti et al., 2020; Rasch et al., 2009). Furthermore, in genetically predisposed individuals, statins can induce HMG-CoA reductase overexpression, triggering an autoimmune response linked to immune-mediated necrotizing myopathy (IMNM) onset (Mammen, 2016). These patients primarily exhibit muscular symptoms, while rash and other extramuscular manifestations are uncommon. Occasionally, patients with statin-induced IMNM develop a typical DM rash (Vasconcelos and Campbell, 2004). According to the latest guidelines, a diagnosis of anti-HMGCR IMNM can be confirmed when patients display proximal muscle weakness, elevated CK levels, and positive anti-HMGCR antibodies. However, in cases where atypical clinical manifestations are observed or the phenotype does not align with autoantibody specificity, a tissue biopsy should be conducted to confirm the diagnosis. This underscores the crucial role of tissue biopsy in achieving an accurate diagnosis and highlights potential limitations in the study, which may arise from diagnostic discrepancies due to the evolving classifications of idiopathic inflammatory myopathies and the subjective nature of reports recorded in the FAERS database.

In this study, immune checkpoint inhibitors (ICI) emerged as the second most frequently reported class of drugs. Studies indicate that ICIs target PD-1 & CTLA-4 to disrupt inhibitory signals, activating cytotoxic CD4+/CD8+ T cells. This triggers keratinocyte apoptosis, basal cell inflammation, MHC I upregulation in myofibers, reversing immune evasion and boosting immune cell killing of tumors (Quach et al., 2021). However, ICIs may induce skin/mucosal toxicities, activating T cells against shared antigens, boosting Th1/Th17, releasing IL-17A/IL-22, causing neutrophil/keratinocyte proliferation or hypersensitivity (Tsiogka et al., 2021; Watanabe and Yamaguchi, 2023). This may damage muscles, triggering DM onset. Furthermore, studies have confirmed that DM can occur during the first ICI cycle, with nivolumab having the most reports, followed by pembrolizumab, atezolizumab, and ipilimumab. The first three are anti-PD-1/PD-L1, while ipilimumab is CTLA-4 therapy. PD-1 on T-cells activates immune responses against infected/cancerous cells (Guerra et al., 2023). PD-L1 on tumors inhibits T-cells, aiding evasion. Anti-PD-1/PD-L1 therapies block this, restoring T-cell responses (Krathen et al., 2008). Overly robust immune response may cause immune-related AEs, including autoimmune DM. CTLA-4 regulates T cell maturation/activation. Blocking it enhances T cell response, boosting anti-tumor effects but also autoreactive T cells, which may cause DM (Sibaud, 2018). In addition, ICI-induced immune responses disrupt cytokine balance, raising pro-inflammatory cytokines and lowering anti-inflammatory ones, worsening inflammation in muscles and skin.

Study finds 60 DM cases linked to antimetabolite therapy, with 29 to capecitabine and 14 to hydroxyurea. Regarding its pathogenesis, capecitabine, by converting to 5-fluorouracil via thymidylate synthase in cancer cells, inhibits DNA synthesis and proliferation, disrupting neovascularization, activating immune cells, and inducing apoptosis. This may elicit autoimmune reactions, contributing to DM onset, especially in the context of high tumor metabolism and antigen release (Chen et al., 2014; Mammen, 2011). Among DIDM, Hydroxyurea is frequently mentioned. DM induced by Hydroxyurea may emerge after long-term (2–10 years) continuous treatment (Martorell-Calatayud et al., 2009). Studies suggest hydroxyurea-induced DM involves chronic, cumulative cytological damage at the epidermal basal layer, due to its inhibition of DNA synthesis/repair, causing cellular dysfunction (Zappala et al., 2012). Additionally, hydroxyurea stimulates local cytokine production (IL-1, IL-8, GM-CSF), exacerbating inflammatory cascades and DM pathology (Oskay et al., 2002). Stopping hydroxyurea controls adverse reactions, with improvement in 1–12 months, often non-recurring (Senet et al., 1995).

Furthermore, a total of 56 DM cases have been linked to taxanes (Paclitaxel, Docetaxel). The mechanism of taxanes operates by inhibiting microtubule disassembly, halting mitosis, arresting of the cell cycle at the G2/M phase and triggering the activation of cellular apoptosis pathways (Alamón-Reig et al., 2021). The precise mechanism of taxane-induced DM remains elusive, potentially involving Taxane-induced apoptosis in keratinocytes that may prompt nucleosome release, and its microtubule-stabilizing effect during mitotic inhibition facilitating Ro52 antigen exposure to the immune system, ultimately eliciting a secondary local autoimmune response. It may be triggered by the drug or its solvent, polyoxyethylated castor oil, which has the capability to elicit complement activation, subsequently leading to hypersensitivity reactions and autoimmune manifestations (Marupudi et al., 2007). Additionally, taxanes was classified as irritants, may induce “vesicant” reactions following extravasation into soft tissues, potentially causing muscle necrosis or chronic ulceration with higher drug volumes or concentrations (Sibaud et al., 2016).

Monoclonal antibodies are also reported to induce DM, including 36 cases of Trastuzumab. Trastuzumab, as an antibody, binds to Fc receptors on immune cells to modulate the immune response. In the presence of cancer cells overexpressing HER2, trastuzumab opsonizes these cells and subsequently engages with CD16 on natural killer (NK) cells, stimulating their elimination of the cancer cells through CD16-mediated antibody-dependent cellular cytotoxicity (ADCC). However, if errors occur during this process, normal skin and muscle cells may also be targeted, potentially leading to the development of DM (Trontzas et al., 2021; Vu and Claret, 2012).

Apart from that, there are also reports on the DM induced by Tetracyclines (n = 24, such as Minocycline and Doxycycline), Platinum Compounds (n = 23, such as Carboplatin), Disease-Modifying Antirheumatic Drugs (n = 17, such as Hydroxychloroquine), and Proton Pump Inhibitors (n = 17, such as Omeprazole). Our review showed limited data on DM from the first three drugs, but implicated PPIs, especially Omeprazole, in DM onset. This may be linked to rhabdomyolysis risk, from intracellular disruption and cell breakdown via H/K-ATPases blockade, or autoimmune muscle disease in familial myopathy patients (Sun et al., 2024).

### 4.2 The analysis of adverse event factors and disease factors

Regarding the reporting of countries in our study, the United States accounted for the highest number of cases. Further, a more extensive examination is necessary to discern potential biases in reporting based on geographic location and ethnic background. Among patients with DM, female individuals may exhibit heightened susceptibility, potentially attributable to underlying immunopathogenesis mechanisms. In this study, the indication of rheumatoid arthritis and Dyslipidaemia reported the most cases. This finding is consistent with the high proportion of statins and anti-rheumatic drugs in drug usage, indirectly validating the reliability of the data. It is noteworthy that the relatively high number of indications for rheumatoid arthritis may stem from two potential reasons. Firstly, patients with rheumatoid arthritis, who have been diagnosed by specialized physicians, may develop DM as an induced condition during systemic drug treatment, such as anti-TNF-α therapy (Xue et al., 2016). The disease development may be correlated with the heightened interferon production observed after TNF-α blockade, given the participation of interferon-induced molecules in the pathogenesis of DM (Takata et al., 2018). Additionally, there may also be cases where patients have been either undiagnosed or misdiagnosed with rheumatoid arthritis. This is because Gottron’s papules, one of the typical manifestations of DM, are violaceous in color and located over the dorsal-lateral interphalangeal and metacarpophalangeal joints, which sometimes resemble the location of rheumatoid nodules (Sontheimer et al., 2003). Furthermore, DM can sometimes induce arthritis or arthralgias, present in approximately 25%–50% of adult DM cases and 35%–61% of juvenile DM cases (Levin and Werth, 2006). Consequently, if patients have not undergone examination by specialized physicians or have been misdiagnosed, they may confuse Gottron’s papules and arthritis/arthralgias caused by DM with rheumatoid arthritis, leading to an increased number of reports.

### 4.3 Limitations of the study

It is noteworthy that this study possesses certain limitations. Firstly, the coding for DM cases may not adhere to current classification criteria, potentially resulting in the misclassification of subtypes. Secondly, the retrospective nature of this study’s management prevents the direct inference of a causal relationship between the drug and DM from the outcomes. Furthermore, there is a risk of reporting bias in the data due to the voluntary reporting of AEs, which could lead to either underreporting or overreporting and significantly affect the ROR analysis. Additionally, the reported association between the drug and DM is confounded by the presence of comorbidities and concomitant medications, despite the data analysis solely focusing on the primary suspect drugs. For instance, ICI-induced myositis can be challenging to differentiate from cancer-associated myositis. Moreover, the FAERS database lacks comprehensive patient-level data, which limits the capacity to assess confounding factors and conduct rigorous statistical analyses, such as those related to genetic and environmental backgrounds. Lastly, several supplements that may trigger DM have not been included in the primary data. Several studies have demonstrated that Spirulina platensis, Aphanizomenon flos-aqua, Chlorella, Echinacea, and alfalfa can activate immune cells through specific cytokines and chemokines, potentially inducing DM (Bax et al., 2021).

### 4.4 Research significance

This study contributes to the continuous monitoring of drug safety and the identification of potential safety issues associated with specific drugs by analyzing AEs related to DIDM from the FAERS. Timely interventions can then be implemented to protect patient safety. Furthermore, this research provides insights into the pathogenesis of DIDM and enhances our understanding of the disease’s pathobiology by exploring the relationship between drug exposure and the development of DIDM. The findings of this study can also inform clinical decision-making, particularly in the selection and monitoring of medications that may be associated with DIDM. Clinicians can utilize this information to identify and manage DIDM early, thereby improving patient outcomes. Additionally, by highlighting the potential risks associated with specific drugs, this study raises awareness among patients, healthcare providers, and regulatory agencies regarding drug safety.

Therefore, to further deepen our understanding of the relationship between drugs and DM, future research should adopt more prospective design strategies. For instance, setting clear observation periods will enable systematic tracking of patients’ long-term health changes following drug exposure. Additionally, research efforts should focus on collecting and analyzing more comprehensive and detailed data, which, beyond basic drug usage records and diagnostic information, should also incorporate patients’ genetic backgrounds, lifestyles, environmental factors, and potential co-existing conditions. Lastly, more advanced statistical methods and models can be employed to conduct in-depth data analysis, further validating the research findings.

## 5 Conclusion

This study encompassed 1767 eligible DM cases spanning from January 2004 to June 2024. Through rigorous screening, 353 drugs associated with DM were identified, and an algorithmic approach further narrowed down the list to 24 signal-positive drugs. The results revealed that the most prevalent reporting characteristics among these cases were female gender, age ranging from 18 to 65 years, and a primary indication of rheumatoid arthritis. Geographically, the United States provides the highest number of reports. The most frequently reported drug categories were cardiovascular drugs (specifically statins), closely followed by immunotherapy agents (including immune checkpoint inhibitors), and chemotherapy agents (notably antimetabolites).

In conclusion, drug-induced DM represents a significant immune-related adverse reaction, necessitating prompt and ongoing medication monitoring to prevent unfavorable outcomes. Upon occurrence of drug-induced DM, the management approach should be tailored to the severity of the condition, which may involve observation, dose reduction, discontinuation of the offending drug, and substitution with alternative treatments.

## Data Availability

The original contributions presented in the study are included in the article/Supplementary Material, further inquiries can be directed to the corresponding authors.

## References

[B1] Alamón-Reig F. Rizo-Potau D. Laguna J. C. Fuertes de Vega I. (2021). Paclitaxel-related eruption mimicking dermatomyositis. J. Dtsch. Dermatol Ges. 19 (7), 1074–1075. 10.1111/ddg.14466 33890396

[B2] Alvarado C. M. Marín Sánchez A. Lima Ruiz J. (2015). Statins and autoimmunity. Med. Clin. Barc. 145 (9), 399–403. 10.1016/j.medcli.2014.11.017 25662717

[B3] Bax C. E. Chakka S. Concha J. S. S. Zeidi M. Werth V. P. (2021). The effects of immunostimulatory herbal supplements on autoimmune skin diseases. J. Am. Acad. Dermatol 84 (4), 1051–1058. 10.1016/j.jaad.2020.06.037 32553683 PMC7736300

[B4] Bendewald M. J. Wetter D. A. Li X. Davis M. D. (2010). Incidence of dermatomyositis and clinically amyopathic dermatomyositis: a population-based study in Olmsted County, Minnesota. Arch. Dermatol 146 (1), 26–30. 10.1001/archdermatol.2009.328 20083689 PMC2886726

[B5] Borges I. B. P. Silva M. G. Misse R. G. Shinjo S. K. (2018). Lipid-lowering agent-triggered dermatomyositis and polymyositis: a case series and literature review. Rheumatol. Int. 38 (2), 293–301. 10.1007/s00296-017-3821-3 29027009

[B6] Caravan S. Lopez C. M. Yeh J. E. (2024). Causes and clinical presentation of drug-induced dermatomyositis: a systematic review. JAMA Dermatol 160 (2), 210–217. 10.1001/jamadermatol.2023.5418 38198130

[B7] Chemello R. M. L. Benvegnú A. M. Dallazem L. N. D. Chemello D. (2017). Aggressive and fatal statin-induced dermatomyositis: a case report. Oxf Med. Case Rep. 2017 (12), omx063. 10.1093/omcr/omx063 PMC5726469 29255614

[B8] Chen F. W. Zhou X. Egbert B. M. Swetter S. M. Sarin K. Y. (2014). Dermatomyositis associated with capecitabine in the setting of malignancy. J. Am. Acad. Dermatol 70 (2), e47–e48. 10.1016/j.jaad.2013.10.025 24438983

[B9] DeWane M. E. Waldman R. Lu J. (2020). Dermatomyositis: clinical features and pathogenesis. J. Am. Acad. Dermatol 82 (2), 267–281. 10.1016/j.jaad.2019.06.1309 31279808

[B10] Fania L. Didona D. Tonanzi T. Mazzanti C. Didona B. (2017). Simvastatin-associated dermatomyositis. Dermatol Ther. 30 (4), e12480. 10.1111/dth.12480 28296009

[B11] Fehr T. Kahlert C. Fierz W. Joller-Jemelka H. I. Riesen W. F. Rickli H. (2004). Statin-induced immunomodulatory effects on human T cells *in vivo* . Atherosclerosis 175 (1), 83–90. 10.1016/j.atherosclerosis.2004.02.016 15186950

[B12] Guerra N. L. Matas-García A. Serra-García L. Morgado-Carrasco D. Padrosa J. Aldecoa I. (2023). Dermatomyositis unleashed by immune checkpoint inhibitors. Three additional cases and a review of the literature. Autoimmun. Rev. 22 (8), 103375. 10.1016/j.autrev.2023.103375 37321468 PMC10529928

[B13] Guo J. Wang W. Huang A. Mei C. (2024). Pharmacological strategies in dermatomyositis: current treatments and future directions. Med. Sci. Monit. 30, e944564. 10.12659/MSM.944564 39275800 PMC11409827

[B14] Krathen M. S. Fiorentino D. Werth V. P. (2008). Dermatomyositis. Curr. Dir. Autoimmun. 10, 313–332. 10.1159/000131751 18460893 PMC3928019

[B15] Kronzer V. L. Kimbrough B. A. Crowson C. S. Davis J. M. 3rd Holmqvist M. Ernste F. C. (2023). Incidence, prevalence, and mortality of dermatomyositis: a population-based cohort study. Arthritis Care Res. Hob. 75 (2), 348–355. 10.1002/acr.24786 PMC8934743 34549549

[B16] Levin J. Werth V. P. (2006). Skin disorders with arthritis. Best. Pract. Res. Clin. Rheumatol. 20 (4), 809–826. 10.1016/j.berh.2006.05.001 16979539

[B17] Luo Y. B. Mastaglia F. L. (2015). Dermatomyositis, polymyositis and immune-mediated necrotising myopathies. Biochim. Biophys. Acta 1852 (4), 622–632. 10.1016/j.bbadis.2014.05.034 24907561

[B18] Mammen A. L. (2011). Autoimmune myopathies: autoantibodies, phenotypes and pathogenesis. Nat. Rev. Neurol. 7 (6), 343–354. 10.1038/nrneurol.2011.63 21654717

[B19] Mammen A. L. (2016). Statin-associated autoimmune myopathy. N. Engl. J. Med. 374 (7), 664–669. 10.1056/NEJMra1515161 26886523

[B20] Martorell-Calatayud A. Requena C. Nagore-Enguídanos E. Guillén-Barona C. (2009). Multiple, painful, treatment-resistant leg ulcers associated with dermatomyositis-like lesions over the interphalangeal joints induced by hydroxyurea. Actas Dermosifiliogr. 100 (9), 804–807. 10.1016/s1578-2190(09)70176-3 19889302

[B21] Marupudi N. I. Han J. E. Li K. W. Renard V. M. Tyler B. M. Brem H. (2007). Paclitaxel: a review of adverse toxicities and novel delivery strategies. Expert Opin. Drug Saf. 6 (5), 609–621. 10.1517/14740338.6.5.609 17877447

[B22] McKee S. Xenakis J. Makin H. Marshall C. Winnette R. Aggarwal R. (2024). Cutaneous manifestations in patients with dermatomyositis, are they only skin deep? Dermatol Ther. (Heidelb). 14 (10), 2771–2785. 10.1007/s13555-024-01266-1 39264400 PMC11480284

[B23] Noël B. (2007). Lupus erythematosus and other autoimmune diseases related to statin therapy: a systematic review. J. Eur. Acad. Dermatol Venereol. 21 (1), 17–24. 10.1111/j.1468-3083.2006.01838.x 17207162

[B24] Oskay T. Kutluay L. Ozyilkan O. (2002). Dermatomyositis-like eruption after long-term hydroxyurea therapy for polycythemia vera. Eur. J. Dermatol 12 (6), 586–588. 12459535

[B25] Quach H. T. Johnson D. B. LeBoeuf N. R. Zwerner J. P. Dewan A. K. (2021). Cutaneous adverse events caused by immune checkpoint inhibitors. J. Am. Acad. Dermatol 85 (4), 956–966. 10.1016/j.jaad.2020.09.054 34332798

[B26] Rasch A. Schimmer M. Sander C. A. (2009). Simvastatin-induzierte dermatomyositis [Simvastatin-induced dermatomyositis]. Hautarzt 60 (6), 489–493. German. 10.1007/s00105-008-1637-5 18853127

[B27] Seidler A. M. Gottlieb A. B. (2008). Dermatomyositis induced by drug therapy: a review of case reports. J. Am. Acad. Dermatol. 59 (5), 872–880. 10.1016/j.jaad.2008.05.034 18625537

[B28] Senet P. Aractingi S. Porneuf M. Perrin P. Duterque M. (1995). Hydroxyurea-induced dermatomyositis-like eruption. Br. J. Dermatol 133 (3), 455–459. 10.1111/j.1365-2133.1995.tb02677.x 8547004

[B29] Sibaud V. (2018). Dermatologic reactions to immune checkpoint inhibitors: skin toxicities and immunotherapy. Am. J. Clin. Dermatol 19 (3), 345–361. 10.1007/s40257-017-0336-3 29256113

[B30] Sibaud V. Lebœuf N. R. Roche H. Belum V. R. Gladieff L. Deslandres M. (2016). Dermatological adverse events with taxane chemotherapy. Eur. J. Dermatol 26 (5), 427–443. 10.1684/ejd.2016.2833 27550571 PMC5526115

[B31] Solanki V. G. Zambare U. S. Dongre A. M. Nayak C. S. (2024). Penicillamine-induced juvenile dermatomyositis: a rare clinical encounter. Indian Dermatol Online J. 15 (5), 851–853. 10.4103/idoj.idoj_657_23 39359283 PMC11444453

[B32] Sontheimer R. D. Costner M. I. (2003). “Dermatomyositis,” in Fitzpatrick’s dermatology in general medicine. Editors Freedberg I. M. Eisen A. Z. Wolff K. Austen K. F. Goldsmith L. A. Katz S. I. 6th edn. (New York: McGraw-Hill).

[B33] Sun X. Zhang C. Jin H. Sun G. Tian Y. Shi W. (2016). Flow cytometric analysis of T lymphocyte proliferation *in vivo* by EdU incorporation. Int. Immunopharmacol. 41, 56–65. 10.1016/j.intimp.2016.10.019 27816727

[B34] Sun Y. Zhang A. Zuo M. Chen J. Zhu L. (2024). A pharmacovigilance study of association between proton-pump inhibitors and rhabdomyolysis event based on FAERS database. J. Gastroenterol. Hepatol. 39 (2), 289–296. 10.1111/jgh.16411 37961012

[B35] Sunderkötter C. Nast A. Worm M. Dengler R. Dörner T. Ganter H. (2016). Guidelines on dermatomyositis--excerpt from the interdisciplinary S2k guidelines on myositis syndromes by the German Society of Neurology. J. Dtsch. Dermatol Ges. 14 (3), 321–338. 10.1111/ddg.12909 26972210

[B36] Takata M. Yamasaki A. Yamada N. Hagino H. Funaki Y. Harada T. (2018). A case of clinically amyopathic dermatomyositis that developed during anti-TNF-α therapy for rheumatoid arthritis. Allergol. Int. 67 (2), 286–288. 10.1016/j.alit.2017.09.001 28964642

[B37] Trontzas I. P. Syrigos N. K. Kotteas E. A. (2021). A case of trastuzumab-induced dermatomyositis. J. Cancer Res. Ther. 17 (4), 1112–1114. 10.4103/jcrt.JCRT_209_19 34528573

[B38] Tsiogka A. Bauer J. W. Patsatsi A. (2021). Bullous pemphigoid associated with anti-programmed cell death protein 1 and anti-programmed cell death ligand 1 therapy: a review of the literature. Acta Derm. Venereol. 101 (1), adv00377. 10.2340/00015555-3740 33426566 PMC9309843

[B39] Vasconcelos O. M. Campbell W. W. (2004). Dermatomyositis-like syndrome and HMG-CoA reductase inhibitor (statin) intake. Muscle Nerve 30 (6), 803–807. 10.1002/mus.20127 15389654

[B40] Visconti M. J. Bashyam A. M. Jorizzo J. L. (2020). Statin-induced dermatomyositis for the practicing dermatologist: a review of the literature. Int. J. Dermatol 59 (3), 383–387. 10.1111/ijd.14751 31840246

[B41] Vu T. Claret F. X. (2012). Trastuzumab: updated mechanisms of action and resistance in breast cancer. Front. Oncol. 2, 62. 10.3389/fonc.2012.00062 22720269 PMC3376449

[B42] Watanabe T. Yamaguchi Y. (2023). Cutaneous manifestations associated with immune checkpoint inhibitors. Front. Immunol. 14, 1071983. 10.3389/fimmu.2023.1071983 36891313 PMC9986601

[B43] Xue Y. Cohen J. M. Wright N. A. Merola J. F. (2016). Skin signs of rheumatoid arthritis and its therapy-induced cutaneous side effects. Am. J. Clin. Dermatol 17 (2), 147–162. 10.1007/s40257-015-0167-z 26649439

[B44] Yu R. J. Krantz M. S. Phillips E. J. Stone C. A. Jr (2021). Emerging causes of drug-induced anaphylaxis: a review of anaphylaxis-associated reports in the FDA adverse event reporting system (FAERS). J. Allergy Clin. Immunol. Pract. 9 (2), 819–829.e2. 10.1016/j.jaip.2020.09.021 32992044 PMC7870524

[B45] Zappala T. M. Rodins K. Muir J. (2012). Hydroxyurea induced dermatomyositis-like eruption. Australas. J. Dermatol 53 (3), e58–e60. 10.1111/j.1440-0960.2011.00774.x 22881475

[B46] Zhao X. Si S. (2023). Five genes as diagnostic biomarkers of dermatomyositis and their correlation with immune cell infiltration. Front. Immunol. 14, 1053099. 10.3389/fimmu.2023.1053099 36742332 PMC9889851

